# Are good intentions putting the vaccination ecosystem at risk?

**DOI:** 10.1080/21645515.2016.1172162

**Published:** 2016-06-06

**Authors:** Michael Watson, Eliot Faron de Goër

**Affiliations:** a Valera Vaccines, Cambridge, MA, USA; b Vaccination Policy and Advocacy, Sanofi Pasteur, Lyon, France

**Keywords:** economics, funding, health policy, non-governmental organization, vaccination program

## Abstract

Vaccination is made possible by an interconnected and interdependent ecosystem of vaccine producers, vaccination policy makers and implementers, and vaccine procurers and funders. The future of vaccination depends on the continued health of this ecosystem and its ability to produce, purchase, deliver, and innovate. However, the number of vaccine producers that also do significant research and development has decreased over the last several years. Many of these R&D-based producers have been forced to cease production of critical vaccines, despite global shortages, so that in several cases only one or two producers remain. We discuss the reasons for these changes and what might be done to maintain a healthy vaccination ecosystem.

## The vaccination ecosystem

Vaccination prevents five premature deaths every minute and has been pivotal in the 49% decrease in mortality for children under five years of age between 1990 and 2013.^1^ Improving access to vaccines, vaccination coverage, and sustainability of vaccination has the potential to save even more lives^2^ but will require a healthy “vaccination ecosystem” in which innovation and the ability to produce, purchase, and deliver vaccines and vaccination can continue.

**Figure 1.**
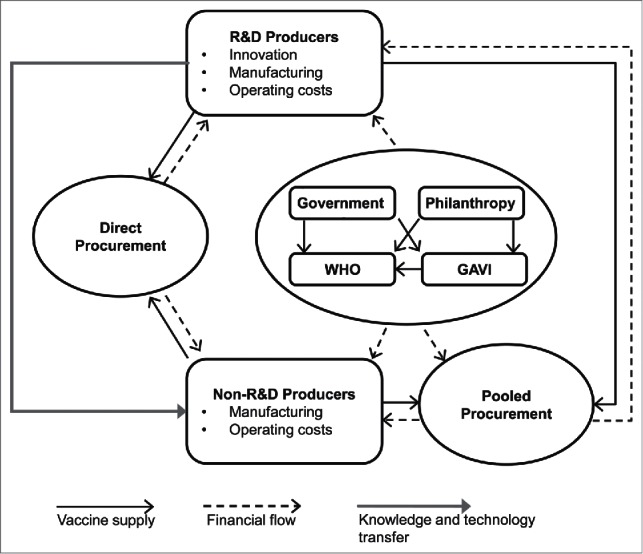
The vaccination ecosystem.

The vaccination ecosystem is an interconnected and interdependent network of vaccine producers, vaccination policy makers and implementers, and vaccine procurers and funders (Fig. 1).^3^ Within the ecosystem are two types of producers: research and development (R&D)-based, which are usually located in high-income countries and invest heavily in developing new vaccines or updating existing ones; and non-R&D-based producers, which manufacture vaccines developed by others, invest little in R&D and scientific expertise, and are often based in low- and middle-income countries.^4,5^ The vaccination ecosystem also includes two, equally important, types of vaccine procurers: direct procurers, which are mostly high- and middle-income countries that define and implement their own vaccination policies and provide their own funding; and pooled procurers, which are usually low- and middle-income countries that pool resources to purchase vaccines through a process usually supported by subsidies.

## Role of the global alliance for vaccines and immunization (GAVI) in the vaccination ecosystem

Prior to the advent of GAVI in 2000, new vaccines were targeted at higher-income countries because they could afford the prices that are needed to recuperate R&D and production costs. Low- and middle-income countries could not access many vaccines, especially new ones, because production volumes were low and prices were beyond their means. GAVI created a mechanism to address this low-income market failure through pooled purchase of vaccines and vaccination support paid for by donations, most notably from the Bill and Melinda Gates Foundation and national donors.^6-8^ The intent was to give producers the confidence to invest in producing higher volumes of vaccines at consequently lower costs and to develop new vaccines better adapted to low- and middle-income countries, such as the pentavalent diphtheria-tetanus-pertussis (DTP)-Haemophilus influenzae type B-hepatitis B virus vaccine.^9^ This mechanism has been successful not only at increasing volume and reducing prices for low- and middle-income countries but also at accelerating the introduction of innovations from the 1980s and 90s, such as pneumococcal conjugate, rotavirus, and human papillomavirus vaccines.

GAVI acts as a pooled procurer, purchasing vaccines through the United Nations Children's Emergency Fund (UNICEF) for countries with a per-capita gross national income below $1570 US.^10^ This pooled procurement delivers mutually beneficial economies of scale due to a single negotiation, delivery, and invoicing point.9,^11^ GAVI is now the world's largest vaccine procurer, and in 2015, they spent $1.7 billion US on vaccines.^12^ GAVI pays tiered prices, made possible by higher prices paid by higher-income, self-procuring countries.^11,13-17^ For example, since 2001, GAVI has made agreements for tiered pricing of rotavirus, human papillomavirus, and pneumococcal conjugate vaccines that are less than one tenth of non-GAVI prices (Table 1).^18-23^

**Table 1.** Prices of vaccines produced by R&D and non-R&D producers.

Vaccine	No. producers				Price/dose (USD)				Price/dose (USD)		
GAVI vaccines	2001^b^	2014	UNICEF Supply status 2014^a^	R&D-producers	2001^b^	2014	Difference^d^	Non-R&D producers	2001^b^	2014	Difference^d^
DTP-HepB-Hib (10 dose vials)	1	3	Supply > demand					Serum Institute of India	1.75–2.105	1.95–2.105	+5.2%
								biological E	—	1.19	
								Panacea	—	1.8–1.94	
DTP-HepB-Hib	1	3	Supply > demand	Crucell/Berna B Korea	3.63	2.4–2.6	−31.1%	biological E	—	2.35	
								Serum Institute of India	—	2.7	
Human papillomavirus	2	2	Supply > demand	GSK	4.6	4.6	0%				
				Merck	4.5	4.5	0%				
Measles (10 dose vials)	3	3	Limited supply	Sanofi Pasteur	0.144	0.45	+212%	Serum Institute of India	0.109	0.252	+131%
								Eisai Co	0.125	—	
								PT Biofarma	—	0.219	
Measles-rubella (10 dose vials)	1	1	Limited supply					Serum Institute of India	0.48	0.55	+15.6%
Meningococcal A (10 dose vials)	1	1	Limited supply					Serum Institute of India	0.426	0.6	+40.8%
Rotavirus	2	2	Very limited supply	GSK	1.88	1.88	0%				
				Merck	5	3.5–5	−15%				
Yellow fever (10 dose vials)	1	2	Very limited supply	Sanofi Pasteur	—	1.133		Institute Pasteur de Dakar	0.5	1.132	+126%
Pneumococcal conjugate vaccine^c^	2	2	Very limited supply	Pfizer	3.5	3.5	0%				
				GSK	3.5	3.5	0%				
Non-GAVI vaccines											
BCG (20 dose vials)	4	3		Statens Serum Institute	0.0615	0.153	+149%	Intervax	0.0466	0.073	+56.7%
				Sanofi Pasteur	0.13	—		Japan BCG Lab	0.0565	0.137	+142%
								Serum Institute of India	—	0.065	
DT (10 dose vials)	2	2						Intervax	0.0585	0.1195	+104%
								PT Biofarma	0.04	—	
								SII	—	0.115	
DTP (10 dose vials)	3	1		Sanofi Pasteur	0.09	—		PT Biofarma	0.066	0.197	+198%
								Serum Institute of India	0.09	—	
Td (10 dose vials)	2	3						Intervax	0.048	0.117	+144%
								Serum Institute of India	0.046	0.11	+139%
								BioFarma	—	0.1	
TT (10 dose vials)	3	4						Intervax	0.0375	0.093	+148%
								Serum Institute of India	0.038	0.077	+103%
								Shanta	—	0.08	
								Biological E	—	0.07	
								PT biofarma	0.034	—	
											
Hepatitis B (10 dose vials)	2	2		Crucell	0.32	0.16	−50%	LG life sciences	0.31	0.173	−44%
Measles-mumps-rubella (10 dose vials)	1	1		GSK	1.85–2.5	—		Serum Institute of India	—	1.025	
tOPV (10 dose vials)	2	2		GSK	0.102	0.18	+76%				
				Sanofi Pasteur	0.0856	0.205	+139%				

*Note.* —, vaccine not supplied by this manufacturer.

a Source http://www.unicef.org/supply/files/Product_Menu_August_2014.pdf.

b Or date of first GAVI purchase.

c Price fixed via Advance Market Commitment.

d Price increases were calculated using average price when the price/dose was provided as a range.

Pan American Health Organization countries also take advantage of this mechanism. They finance and manage their own vaccination programs but may pool procurement through a revolving fund.^24^ Despite relatively high per-capita gross national incomes, they reference price to GAVI prices through their Lowest Price Clause (LPC).^4,11,25^

## Current health of the vaccination ecosystem

Over the last two decades, there have been many positive changes in the vaccination ecosystem. Prices have dropped for some common vaccines, making them more available to low- and middle-income countries. The weighted average price of the DTP-based pentavalent vaccine has decreased by 20–65%, driven by a combination of more suppliers and a shift from single-dose presentations to less expensive multi-dose presentations.^22^ Prices have also dropped 45–59% for the measles-mumps-rubella vaccine and 46–48% for the hepatitis B vaccine.

Unfortunately a number of negative unintended consequences have also emerged. The measles-mumps-rubella vaccine is now supplied by a sole producer (Serum Institute of India, SII) and its price increased in 2014 by 10%, while hepatitis B vaccine is now supplied by two producers, compared to six prior to 2010. Overall, the number of vaccines supplied to GAVI by a single producer has tripled, from two to six, since 2001 (Table 2). Furthermore, in 2014, supplies were limited or very limited for six of eight vaccines procured by GAVI,^26,27^ most notably the DTP, Bacillus Calmette-Guérin, and oral polio vaccines.^26,28,29^

**Table 2.** Vaccine suppliers and products in 2001 and 2014.

Category	Subcategory	2001	2014
UNICEF-awarded vaccine suppliers	Total	13	16
	R&D producers	6	6
	Non-R&D producers	7	10
Vaccines supplied by a single producer	Total	2	6
	R&D producers	2	1
	Non-R&D producers	0	5
	Vaccines	MenPS, DTP-HB-Hib	YF^a^, MR, MenAconj, rabies, DTP, MMR^b^

*Note.* Abbreviations: DTP, diphtheria-tetanus-pertussis vaccine; DTP-HB-Hib, diphtheria-tetanus-pertussis-Haemophilus influenzae type B-hepatitis B virus vaccine; MenAconj, Meningococcal A conjugate vaccine; MenPS, meningococcal polysaccharide vaccine; MMR, measles-mumps-rubella vaccine; MR, measles-rubella vaccine; YF, yellow fever vaccine.

a Only Sanofi Pasteur has been able to supply consistently.

b Sanofi Pasteur production of MMR bulk lots has ceased.

A 2002 report by UNICEF found that between 1998 and 2001, 10 of 14 manufacturers partially or totally stopped producing existing vaccines, and both Baxter and Novartis recently sold their vaccines divisions.^28,33,34^ Thus, from a total of 14 R&D-based vaccine producers in the 1990s, only four remain in 2016.^30-32^

The concern comes from GAVI's short term, static efficiency goal to increase demand and lower prices across all producers without measuring the impact on sustainability. R&D-based producers have high capital and operating costs because they are usually located in high-income countries, because they need to fulfill diverse and evolving regulatory, quality, and commercial demands of multiple markets, and because they invest heavily in developing new vaccines or updating existing ones.^4^ To develop a new vaccine, for example, R&D-based producers spend more than 10 y and more than $1 billion US on R&D.^35,36^ Non-R&D-based producers have a different business model. They have lower costs because they usually manufacture vaccines developed by others, invest minimally in R&D and scientific expertise, and are usually based in low- and middle-income countries.^4,5^ The largest non-R&D producer, SII, re-invests 3.1% of sales in R&D compared to 13.9% for an R&D producer such as Sanofi Pasteur (SP) and although emerging manufacturers spend 4–14% of sales on R&D, the largest amount spent by an emerging non-R&D producer was $6 million US/year compared to approximately $500 million US/year for each of the 4 global R&D-based producers.^37-42^ Thus, the profit margins for R&D-based companies are usually lower than for non-R&D producers (e.g. Twenty-four.5% for an R&D-based producer such as SP vs. 49.5% for a non-R&D-based producer such as SII).

As a consequence of this financial asymmetry, non-R&D-based producers can remain profitable at prices where R&D-based producers cannot. Diminishing profitability has forced some R&D producers to stop producing some vaccines. For example, Crucell stopped producing the yellow fever vaccine, Sanofi Pasteur stopped producing measles-containing vaccines, and both CSL and Sanofi Pasteur stopped producing the DTP vaccine, despite global shortages of all three vaccines.^4,9,11^ This loss of vaccine producers leaves the global vaccine supply at risk because of the cost and time needed to scale up when a shortage or outbreak occurs. This leads to a situation where demand can exceed supply, resulting in price increases. For example, since 2001, prices increased 200% for the DTP vaccine, 530% for the yellow fever vaccine, and 25–150% for measles-containing vaccines.^4,9,11^

Lower prices, and therefore lower profitability for R&D-based producers, also reduces the incentives and increases the risks of investing in vaccine R&D. This may result in reduced development of vaccines for countries supplied by GAVI, as well as a re-concentration of R&D in and for high-income countries. A push toward the lowest possible vaccine prices may also result in the loss of vaccine producers in low- and middle-income countries supplied by GAVI. Ironically, the same upper-income countries who are funding GAVI may lose their internal producers, leaving them reliant on producers in low- and middle-income countries.

Survival options in the face of lower profit margins include consolidation. This happened recently with the absorption of Novartis Vaccines by GlaxoSmithKline, leaving only 4 global R&D-based producers (GlaxoSmithKline, Sanofi Pasteur, Merck, and Pfizer). The growing oligopoly is therefore not a business strategy but a response to market forces. Of these four, only two produce human papillomavirus, meningococcal B, and rotavirus vaccines and can supply significant quantities of pentavalent and hexavalent acellular pertussis-containing combination vaccines. Although the four remaining R&D-based vaccine producers have annual vaccine R&D budgets of over $200 million each.^37-39,41^ development of new vaccines may be compromised because the costs of new vaccine development often exceed $1 billion US.

## Recommendations

Philanthropic funding over the last decade or so has been pivotal in increasing access to vaccination for the lowest income countries. Removal of these subsidies would have far-reaching negative effects on the entire ecosystem and is not desirable. However, the ecosystem must not become over-dependent on subsidies, and the sustainability of the vaccination ecosystem must not be compromised to achieve short-term goals. Vaccine manufacturing capabilities and capacities need to be broadened globally but not at the cost of losing manufacturing capabilities and capacities in upper-income countries.

Below, we make two recommendations that should help maintain a healthy and viable vaccination ecosystem.

### Recommendation 1: switch from the current static price-driven efficiency model to a dynamic efficiency model

Although price is a convenient and attractive metric for donors and politicians, it does not reflect the complex realities of the vaccination ecosystem. A price-driven efficiency model is also not aligned with the Global Vaccine Action Plan, which calls for improvement in country ownership, shared responsibility, equity, integration of immunization systems, sustainability, and innovation.^43^ The recent, unintended adverse consequences of this static efficiency model on vaccine supply, price, and number of producers are warning signs. To avoid market failure akin to that seen in antibiotics, the true interrelationships and dynamics of the vaccination ecosystem must be recognized and the ecosystem stewarded accordingly.^44^

Many of the unintended consequences of an ecosystem driven by cost alone could be addressed by switching to a dynamic efficiency approach^43,45^ in which quality, supply reliability, vaccination coverage, and future innovation are also included. Such a vision already exists in the form of the World Health Organization's Global Vaccine Action Plan, although 6 y into the decade of vaccine, appropriate companion metrics do not yet exist.^43^

The alternative would be to continue the current ecosystem but with the risk of reducing donations from governments and philanthropists. This is an undesirable but real possibility due to changing global health priorities, changing political leadership, donor fatigue, and adverse economic conditions. This would return us to the pre-GAVI era which is not desirable. In the absence of sufficient third-party subsidies, technology transfer to non-R&D producers would be less attractive and unsubsidized non-research producers would likely become less viable. This highlights the implicit risks of overdependence on subsidies and the importance of GAVI's graduation policy, whereby countries raise their financial contribution as their per-capita gross national income increases. This also emphasizes the need to build a vaccination ecosystem that is sustainable, incentive-based, and driven and measured by public health objectives in place of the current system, which is shaped largely by the drive for lower prices.^11,46,47^

### Recommendation 2: include representatives from vaccine producers and experts in economics and market dynamics in global policymaking bodies

Public health policy makers, influencers, funders, and financers need to be made aware of the dynamics, strengths, and weaknesses of the vaccination ecosystem. In addition, the vaccination community needs to focus on shared, short- and long-term goals for the ecosystem, which should be aligned with the Global Vaccine Action Plan. Coordinated actions, incentives, and metrics that are enacted and managed by true cross-partner collaborations are needed.

These objectives can be accomplished by including representatives from vaccine producers and experts in economics and market dynamics in global policymaking bodies such as the World Health Organization's Strategic Advisory Group of Experts. Unfortunately, such individuals are often excluded because of perceived conflicts of interest. In reality, they are key components of the vaccination ecosystem and it is hard to see how the vaccination ecosystem can remain healthy if overseen only by experts in public health and by excluding those who understand the functioning of healthy markets and the challenges of developing and producing vaccines.

## Abbreviations

DTP: diphtheria-tetanus-pertussis
GAVI: Global Alliance for Vaccines and Immunization
R&D: research and development
UNICEF: United Nations Children's Emergency Fund

## References

[1] United Nations Inter-agency Group for Child Mortality Estimation Levels & Trends in Child Mortality New York: United Nations Children's Fund, 2014 Available from: http://www.unicef.org/media/files/Levels_and_Trends_in_Child_Mortality_2014.pdf 10.1371/journal.pone.0101112 PMC4094389 25013954

[2] WHO/UNICEF/World Bank State of the World's Vaccines and Immunization, 3rd edition. Geneva: World Health Organization, 2009 Available from: http://apps.who.int/iris/bitstream/10665/44169/1/9789241563864_eng.pdf

[3] Labiotech Eu Who is the Leader in the Vaccination Ecosystem – is it (still) Europe?, 2015 Available from: http://labiotech.eu/who-is-the-leader-in-the-vaccination-ecosystem-europe/

[4] Wilson P Giving developing countries the best shot: An overview of vaccine access and R&D Geneva: Oxfam International, 2010 Available from: https://www.msf.org.uk/sites/uk/files/Vaccine_Report_201005111518.pdf

[5] Mercer Management Consulting Lessons Learned: New Procurement Strategies for Vaccines – Final Report to the GAVI Board Geneva: GAVI Alliance, 2002 Available from: http://www.gavi.org/library/gavi-documents/supply-procurement/mercer-report-on-vaccine-procurement/

[6] GAVI Alliance Gavi's mission, 2015 Available from: http://www.gavi.org/about/mission/

[7] Chee G, Molldrem V, Hsi N, Chankova S Evaluation of GAVI Phase 1 Performance Bethesda, MD: Abt Associates Inc., 2008. Available from:

[8] GAVI Alliance Key figures: donor contributions & pledges, 2016 Available from: http://www.gavi.org/funding/donor-contributions-pledges/

[9] Frontières MS The Right Shot: Extending the Reach of Affordable and Adapted Vaccines Lausanne, Switzerland: Médecins Sans Frontières, 2012 Available from: http://www.msf.org.uk/sites/uk/files/Vaccines__the_Right_Shot_May_2012_201205155725.pdf

[10] GAVI Alliance Country eligibility policy, 2016 Available from: http://www.gavi.org/about/governance/programme-policies/country-eligibility/

[11] Brenzel L, Jones A Immunization Financing and Sustainability Task Team. Immunization Financing Toolkit. A Resource for Policy Makers and Program Managers Geneva: GAVI Alliance, 2010 Available from: http://siteresources.worldbank.org/HEALTHNUTRITIONANDPOPULATION/Resources/281627-1292531888900/IMMUNIZATIONFINANCINGTOOLKITFINAL121410.pdf

[12] GAVI Alliance Report to the Board, 10–11 June 2015 Geneva: GAVI Alliance, 2015 Available from: http://www.gavi.org/library/minutes/gavi-alliance-board/year/2015/

[13] International Federation of Pharmaceutical Manufacturers & Associations Vaccine industry commitment to global access, innovation and sustainability. The role of tiered pricing for vaccines across countries Geneva: International Federation of Pharmaceutical Manufacturers & Associations, 2013 Available from: http://www.ifpma.org/fileadmin/content/Global%20Health/Vaccines/Vac123-F_20130904_IFPMA_Position_on_tiered_pricing_for_vaccines.pdf

[14] Danzon PM, Towse A. Differential pricing for pharmaceuticals: reconciling access, R&D and patents. Int J Health Care Finance Economics 2003; 3:183–205; PMID:14625999; http://dx.doi.org/10.1023/A:1025384819575 14625999

[15] Plahte J. Tiered pricing of vaccines: a win-win-win situation, not a subsidy. Lancet Infect Dis 2005; 5:58–63; PMID:15620562; http://dx.doi.org/10.1016/S1473-3099(04)01255-1 15620562

[16] Moon S, Jambert E, Childs M, von Schoen-Angerer T. A win-win solution?: A critical analysis of tiered pricing to improve access to medicines in developing countries. Globalization Health 2011; 7:39; PMID:21992405; http://dx.doi.org/10.1186/1744-8603-7-39 21992405 PMC3214768

[17] Balasegaram M. Is tiered pricing the way for vaccines? Lancet 2014; 384:852; PMID:25209481; http://dx.doi.org/10.1016/S0140-6736(14)61483-5 25209481

[18] Gilchrist SA, Nanni A. Lessons learned in shaping vaccine markets in low-income countries: a review of the vaccine market segment supported by the GAVI Alliance. Health Policy Planning 2013; 28:838–46; PMID:23174880; http://dx.doi.org/10.1093/heapol/czs123 23174880

[19] UNICEF Supplies and Logistsics. Vaccine Price Data, 2015 Available from: http://www.unicef.org/supply/index_57476.html

[20] Program CfDCaPVfC CDC Vaccine Price List, 2015 Available from: http://www.cdc.gov/vaccines/programs/vfc/awardees/vaccine-management/price-list/

[21] GAVI Alliance About the Pneumococcal AMC, 2015 Available from: http://www.gavi.org/funding/pneumococcal-amc/about/

[22] UNICEF Market updates. Vaccine manufacturer consultation 10 2014, Copenhagen [slide presentation]. New York: UNICEF: UNICEF, 2014. Available from:http://www.unicef.org/supply/files/4.Market_updates.pdf

[23] UNICEF Pneumococcal Vaccine New York: UNICEF, 2015 Available from: http://www.unicef.org/supply/files/PCV.pdf

[24] Pan American Health Organization PAHO Revolving Fund, 2015 Available from: http://www.paho.org/hq/index.php?option=com_content&view=article&id=1864&Itemid=40713&lang=en

[25] Pan American Health Organization PAHO, the Pan American Health Organization Revolving Fund for Vaccine Procurement [Provisional Agenda Item 4.17. CE144/22, Rev. One (Eng.) 11 June 2009]. Washington, DC: Pan American Health Organization, 2009 Available from: http://iris.paho.org/xmlui/bitstream/handle/123456789/4814/CE144-22-e.pdf?sequence=1

[26] UNICEF Product menu for vaccines supplied by UNICEF for the GAVI Alliance New York: UNICEF, 2014 Available from: http://www.unicef.org/supply/files/Product_Menu_August_2014.pdf

[27] UNICEF Supply Division Yellow Fever Vaccine Current Outlook New York: UNICEF, 2014 Available from: http://www.unicef.org/supply/files/YFV_Supply_Status_Update.pdf

[28] UNICEF Vaccines for Children: Supply at Risk New York: UNICEF, 2002 Available from: http://www.unicef.org/publications/files/pub_vaccine_supply_en.pdf

[29] Arnould RJ, DeBrock L An overview of the market for vaccines in the United States Washington, DC: Division of Healthcare Services, Institute of Medicine, 2002 Available from: http://iom.nationalacademies.org/∼/media/Files/Activity%20Files/Disease/VaccineFinancing/ArnouldandDeBrockBackgroundPaper.pdf

[30] Moran M, Guzman J, Chapman N, Abela-Oversteegen L, Howard R, Farrell P, et al. Neglected disease research and development: The public divide. Policy Cures G-FINDER Sydney, Australia: Policy Cures, 2013 Available from: http://www.policycures.org/downloads/GF_report13_all_web.pdf

[31] Ki-moon B, Bokova I, Chambers R, Chopra M, Clark H, Cousin E, et al. Global development goals. Leaving no one behind New York: United Nations Association UK, 2013 Available from: http://www.starsfoundation.org.uk/sites/default/files/downloads/UNA-UK%20Global%20Development%20Goals.pdf

[32] Keith JA, Agostini Bigger L, Arthur PA, Maes E, Daems R. Delivering the promise of the Decade of Vaccines: opportunities and challenges in the development of high quality new vaccines. Vaccine 2013; 31 Suppl 2:B184–93; PMID:23598480; http://dx.doi.org/10.1016/j.vaccine.2012.12.032 23598480

[33] [No authors listed] Baxter Announces Divestiture Commercial Vaccines Business Pfizer 2014 Available from: http://www.businesswire.com/news/home/20140730005367/en#.VBGSfXIQsrR

[34] Helfand C Novartis bids farewell to vaccines with $7.1B sale to GSK Washington DC: FierceVaccines, 2014 Available from: http://www.fiercevaccines.com/story/novartis-bids-farewell-vaccines-71b-sale-gsk/2014-04-22

[35] Philadelphia TCoPo Vaccine development, testing, and regulation, 2014 Available from: http://www.historyofvaccines.org/content/articles/vaccine-development-testing-and-regulation

[36] Garde D Sanofi bets big on dengue with eyes on a blockbuster Washington, DC: FierceBiotech, 2014 Available from: http://www.fiercebiotech.com/story/sanofi-bets-big-dengue-eyes-blockbuster/2014-03-26

[37] [No authors listed] GSK Annual Report 2014 Brentford, UK: GlaxoSmithKline, 2014 Available from: https://www.gsk.com/media/603031/annual-report-2014.pdf

[38] [No authors listed] Appendix A. 2014 Financial Report New York: Pfizer, 2014 Available from: http://www.pfizer.com/system/files/presentation/2014_Pfizer_Financial_Report.pdf

[39] [No authors listed]. Merck & Co., Inc. Annual Report on Form 10-K, Fiscal Year Ended December 31, 2014 New York: United States Securities and Exchange Commission, 2015 Available from:http://phx.corporate-ir.net/External.File?item=UGFyZW50SUQ9Mjc3ODc3fENoaWxkSUQ9LTF8VHlwZT0z&t=1

[40] [No authors listed] Indian Exporters' Excellence Awards - winner available [Internet] Powair, India: Dun & Bradstreet India, 2012 Available from: http://www.dnb.co.in/Exporters2012/Profile%5CSerum%20Institute%20of%20India%20Limited.pdf

[41] [No authors listed] Annual Results 2013 [slide presentation] Paris: Sanofi, 2013 Available from: http://en.sanofi.com/Images/35553_2014-02-06_Results-2013_presentation.pdf

[42] Milstien JB, Kaddar M. The role of emerging manufacturers in access to innovative vaccines of public health importance. Vaccine 2010; 28:2115–21; PMID:20044054; http://dx.doi.org/10.1016/j.vaccine.2009.12.036 20044054

[43] [No authors listed] Global Vaccine Action Plan Monitoring, Evaluation & Accountability, Secretariat Annual Report 2013 Geneva: World Health Organization, 2013 Available from: http://www.who.int/immunization/global_vaccine_action_plan/GVAP_secretariat_report_2013.pdf

[44] Projan S. Why is big Pharma getting out of antibacterial drug discovery? Curr Opin Microbiol 2003; 6:427–30; PMID:14572532; http://dx.doi.org/10.1016/j.mib.2003.08.003 14572532

[45] Abel A, Mankiw G, Summers L, Zeckhauser R Assessing dynamic efficiency: theory and evidence. Rev Economics Statistics 1989; 56:1–20; http://dx.doi.org/10.2307/2297746

[46] United Nations Department of Economic and Social Affairs Monterrey Consensus of the International Conference on Financing for Development, Monterrey, Mexico, 18–22 March 2002 New York: United Nations, 2003 Available from: http://www.un.org/esa/ffd/monterrey/MonterreyConsensus.pdf

[47] [No authors listed] Abuja declaration on HIV/AIDS, tuberculosis, and other related infectious diseases [OAU/SPS/ABUJA/3] African Summit on HIV/AIDS, tuberculosis and other related infectious diseases, Abuja, Nigeria, 24-27 April 2001 Abuja, Nigeria: Organisation of African Unity, 2001 Available from: http://www.un.org/ga/aids/pdf/abuja_declaration.pdf

